# Unusual suicide by a speargun shot: Case report

**DOI:** 10.1097/MD.0000000000022308

**Published:** 2020-12-04

**Authors:** Rosario Barranco, Fiorella Caputo, Sara Lo Pinto, Martina Drommi, Francesco Ventura

**Affiliations:** Department of Forensic and Legal Medicine, University of Genova, Via De’ Toni 12, Genova, Italy.

**Keywords:** autopsy, forensic evaluation, speargun shot, suicide

## Abstract

**Rationale:**

Only a few cases of accidental deaths due to speargun injuries are reported in the literature. Murder or suicide cases are even rarer.

**Patient concerns:**

A 59-year-old male with a history of major depression and previous suicide attempts, was found, still alive and conscious, with a spear in his mouth and a fishing speargun a few meters away. The spear then penetrated the cranium and crossed the entire left cerebral hemisphere.

**Diagnoses:**

The patient underwent a retrograde removal of the spear. During the surgery, there was a massive encephalic bleeding. After about 2 days of coma, brain death was confirmed. An autopsy was performed to determine the cause of death.

**Interventions:**

The scalp presented hemorrhagic infiltrates in the left parieto-temporal region. There were an acute subdural hematoma and subarachnoid hemorrhage. At the opening of the lateral ventricles a massive fronto-parieto-temporal hematoma was evident. The skull base had a massive hemorrhagic infiltration and a circular fracture of about 0.5 cm in diameter, due to the penetration of the spear. The hard palate showed a circular solution of continuity with net margins whose diameter was consistent with the size of the spear.

**Outcomes:**

The cause of death was attributed to the traumatic cranial-encephalic lesions due to the speargun shot in the mouth.

**Lessons:**

The investigation into unusual cases of death constitutes a complex matter and requires a careful evaluation on the part of the forensic pathologist. A differential diagnosis may be necessary in order to rule out simulated suicide/homicide. In this particular case, the analysis of the scene of the self-suppression event and available circumstantial information, the evaluation of clinical data, the complete autopsy and the comparison between the injuries of the victim and the characteristics of the weapon used led to the confirmation of the suicidal nature of the death.

## 1 Introduction

Penetrating injuries to the oral cavity represent common methods of violent suicide.^[1,2]^ This type of injury is usually due to the use of firearms^[2,3]^ whereas cranio-encephalic damage in another way than from a gunshot (example: low velocity penetrating brain injury) is extremely rare.^[4]^

Surely, in the evaluation of the methods and systems used to determine stab wounds, forensic pathologists should focus their attention on cases of violent deaths caused by the use of unusual weapons.^[5]^

Only a few cases of accidental deaths carried out by the use of harpoons, darts, crossbows or spear guns are reported in the literature.^[2,5–14]^

Weapons that fire sharp objects or arrows provide a speed of action that is similar to the objects used in stabbings and possess intrinsic differences in the mechanism of the injury.^[4,15,16]^

In spite of its name, a fishing speargun resembles in shape and function, more like a crossbow or an arch, and, just as these, does not require a gun license, even though it can cause very similar injuries to wounds caused by firearms.

The evaluation of the causes and methods of death in these cases requires a thorough a multidisciplinary evaluation.^[2]^

In the present report, the authors present an unusual case of suicide caused by extensive cranio-brain trauma secondary to a shot fired from a fishing speargun into the mouth. Death was not immediate but it did take place after 2 days of hospitalization.

## 2 Case report

A 59-year-old male, licensed scuba diver, with a history of major depression and previous suicide attempts, was found, still alive and conscious, with a spear in his mouth and a fishing speargun a few meters away.

**Figure 1:**
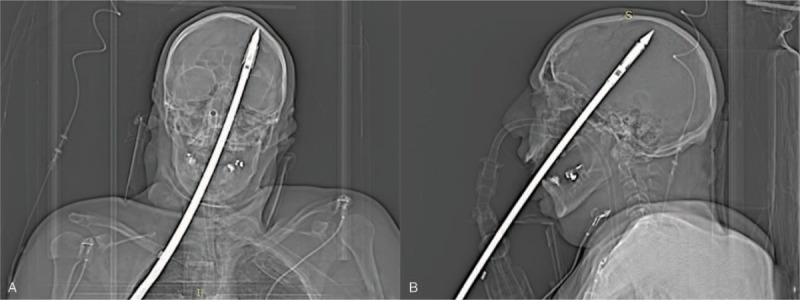
3D CT image reconstruction. The metallic object penetrated the oral cavity and crossed the left hard palate, nasal cavity, pterygoid process, and the sphenoid hemisphere, orbital apex region, orbital fissure, and the left optical channel.

**Figure 2:**
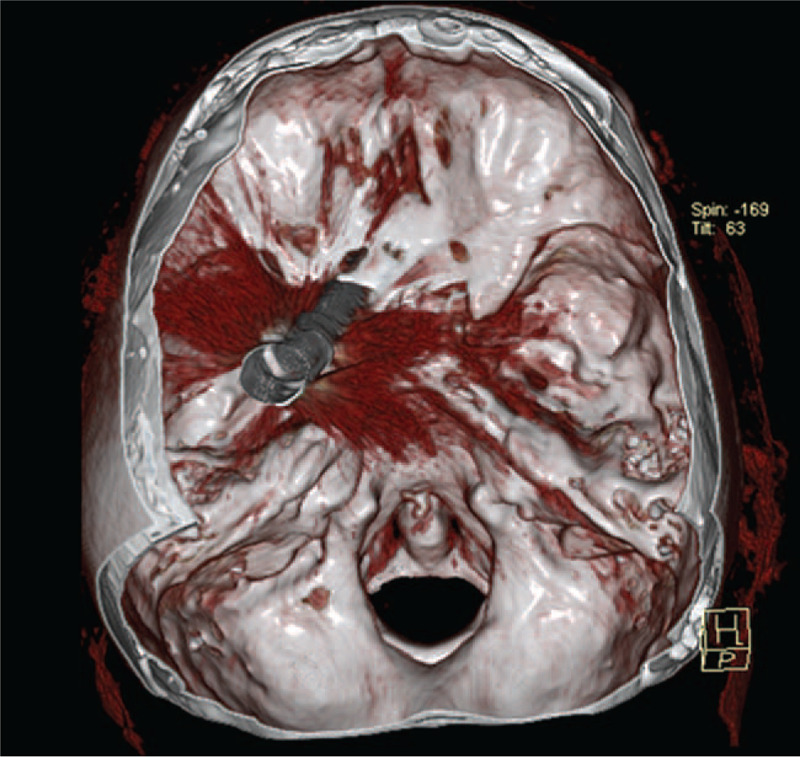
CT images. The metallic object penetrated the cranium and crossed the entire left cerebral hemisphere. The tip of the harpoon was embedded in the left upper parietal cranium, with a displaced fracture and a slight exocranial protrusion of the stumps. (2A Coronal CT scan; 2B Sagittal CT scan).

The subject was brought to a hospital where a performed brain CT (computer tomography) with 3D (three-dimensional) reconstruction identified a metallic rod that had penetrated the oral cavity and crossed the left hard palate, nasal cavity, pterygoid process, and the sphenoid hemisphere, orbital apex region, orbital fissure, and the left optical channel. The spear then penetrated the cranium and crossed the entire left cerebral hemisphere (Figs. 1 and 2). The tip of the harpoon was embedded in the left upper parietal cranium, with a displaced fracture and a slight exocranial protrusion of the stumps.

**Figure 3:**
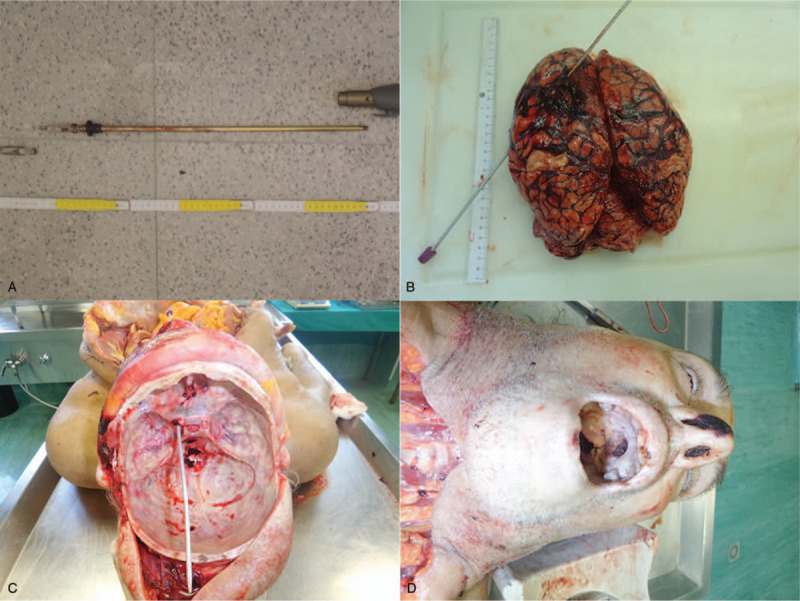
Autopsy findings. A) The spear of the speargun used to commit the suicide. B) The brain showed hemorrhagic infiltrates of the left fronto-parieto-temporal lobes. C) The spear penetrated the cranium and crossed the entire left cerebral hemisphere. D) The hard palate showed a circular solution of continuity with net margins whose diameter was consistent with the size of the spear.

The patient underwent a retrograde removal of the spear (Fig. 3A). However, during the surgery, there was massive and unstoppable encephalic bleeding that involved the fronto-parieto-temporal region. After about 2 days of coma, brain death was confirmed.

An autopsy was performed to determine the cause of death and whether the suicidal ideation was compatible with the injuries sustained, taking into account the nature and size of the fishing speargun found next to the person.

At the skull section, the scalp presented hemorrhagic infiltrates of its deep surface and periosteum in the left parieto-temporal region. The craniotomy (with the open bone flap of about 6 cm in diameter) was present in the parietal region, from which part of the left parietal lobe was herniated. The macroscopic examination showed an acute subdural hematoma and subarachnoid hemorrhage at the left fronto-parieto-temporal lobe (Fig. 3B). The path caused by the harpoon arrow that obliquely crossed the front left hemisphere was evident, from right to left and from downward to upward.

At the opening of the lateral ventricles a massive fronto-parieto-temporal hematoma was evident (close to the *gyrus cinguli*).

The skull base had a massive hemorrhagic infiltration and a circular fracture of about 0.5 cm in diameter, due to the penetration of the spear (Fig. 3C). The hard palate showed a circular solution of continuity with net margins whose diameter was consistent with the size of the spear (Fig. 3D).

The cause of death was attributed to the traumatic cranial-encephalic lesions due to the speargun shot in the mouth.

## 3 Discussion

Speargun is a weapon that can work through 2 different mechanisms: the first is pneumatic compression inside a barrel and the second is the stretching of a particular type of rubber band.

The spread of static energy to the spear through a trigger mechanism causes a sudden acceleration of the spear.^[17]^

Many cases of death due to speargun shots are accidental and happen during water sport activities.^[17–21]^ In other cases, this kind of events are due to lack of attention or non-compliance with the safety rules of underwater fishing.^[22]^

Accidental lesions are generally mild and not complicated. At the same time, however, this kind of injuries can be devasting and fatal if they involve the regions of the skull or the neck.^[19]^

Usually, the sizes of a common speargun can vary in a range from 0.5 to 2 m in length and from 28 to 75 mm in diameter.^[23,24]^

As this type of weapon is projected for underwater fishing, its force must overcome water resistance. If the weapon is used in absence of water resistance, as in the presented case, it can cause a very severe injury. Perforating cranial and facial lesions are associated with a high mortality rate because of the important damage of the parenchyma and the neurovascular structures of the brain.^[23,24]^

Homicide or suicide cases perpetrated by a speargun shot are extremely rare: only 3 cases are described in the Literature.^[2,5,14]^ These cases therefore need a particular attention to confirm the medico-legal diagnosis of homicide, suicide or accident.^[2]^ The more the death scene is atypical, the more the differential diagnosis is complicated.^[25]^

Despite the speargun could be considered as an atypical firearm^[5]^ it is important to consider that the spear shot has a lower kinetic energy compared to a common firearm. Because of the unusual conformation of the spear, the injuries caused by this kind of weapon have unique features. In high speed penetrating trauma, the great kinetic energy causes a radial force which determine a massive destruction of tissues.

Low speed weapons, such as speargun, have a lower kinetic energy so they cause milder injuries that are often limited to the spear pathway.^[4]^

Moreover, it is necessary to consider that the distinctive feature of speargun is the presence of barb at the extremity of the spear. Consequently the main tissue damage in this kind of injuries can be obtained just in the attempt to extract the spear.^[17]^

In speargun injuries it is important to set the course and the final point of the spear through an accurate radiological investigation to determine the best surgical approach for its removal; because of the rarity of this type of injury there are no surgical guidelines for the treatment of these lesions.^[4]^

As a rule, the choice of method in committing suicide depends on access to harm-inflicting means, sociocultural circumstances and individual characteristics (age, sex, and subjects personality).^[2,26]^ As confirmed by the instance under scrutiny, the subjects hobbies play a significant role in the mode of committing suicide.^[2,27,28]^ Indeed, in the case presented the victim was an underwater fishing enthusiast. Some authors have offered criteria to distinguish between usual or unusual suicide.^[26,29]^ As a matter of fact, suicide cases may be considered atypical in the light of the following: dynamics and type of lesion, methods employed (originality, statistical frequency, complexity, compatibility with the cause of death), examination of the location of death and lack of evidence indicating a psychological disorder.^[28–30]^ In the determining of a score for each of the criteria mentioned, some authors^[26]^ have suggested that unusual suicide cases should be classified as slightly atypical, partially atypical and decidedly atypical.

The anatomic sites pertaining to suicide wounds are in general easy to reach - except in the cases where a device is intentionally introduced (for example, the use of elastics) so as to self-inflict some distance from actual suicide location.^[2]^

As to the choice of anatomic site, the suicide often selects to draw on tried and tested areas such as the temples, the mouth, the neck, the chin, the precordial region of the thorax and the abdomen.^[31,32]^ A history of psychiatric disorders is often present in suicide cases.^[2]^

Penetrating lesions to the skull and face are linked to a high mortality rate, since they cause serious damage to the parenchyma and neurovascular structures of the brain.^[23,33]^ Moreover, a shot fired into the oral cavity through the cerebral trunk results in an immediately lethal lesion.^[2,32,34]^ In other cases described in literature,^[6,22]^ reference is made to a survival period pursuant to perforating brain injury.

In the suicide in question, death was not immediate – this because the harpoon fired did not go through the encephalic trunk and, thus, the cardio-respiratory centres were not immediately involved. The progressive worsening of the encephalic damage and intra cranial haemorrhage (also resulting from neurosurgical efforts to extract the harpoon) brought on a massive increase in endo-cranial pressure as well as hypoxic brain damage spreading to the diencephalon and encephalic trunk.

In this specific case, the differential diagnosis between simulated suicide/homicide and suicide has been possible through an analysis of the characteristics of the lesions, the type of weapon employed and circumstantial details.

The underwater rifle was, for the record, 40 cm long, as was the harpoon shaft impaled in the oral cavity (length 40 cm and diameter 8 cm). The harpoon measured about 15 cm. Comparing such measurements with the length of the victims arms (bilaterally 86 cm from the shoulder to the fingertips), the same measurements were compatible with self-infliction dynamics: the subject was seated on a chair at the time of harpoon release. After release, the rifle made a pressure-induced, reverse movement towards the door of the garage – this displacing the weapon some meters from the victim and, thus, explaining the distance between the subject and the weapon employed.

It is to be added that the victim did not present hypothetical signs of a scuffle or indications of an attempt in self-defence. The selected region too (oral cavity) constitutes a typical suicidal choice of site on the human anatomy. Finally, the analysis of circumstantial and medical history details has revealed that he was subject to major depression and that he had previously attempted to take his life.

## 4 Conclusions

The investigation into unusual cases of death, indeed, constitutes a complex matter and requires a careful evaluation on the part of the forensic pathologist. The correct differential diagnosis between homicide and suicide demands a careful analysis of the scene of the death, a thorough post mortem, accurate evaluation of circumstantial medical history details and a comparison between lesions inflicted on/by the victim and the characteristics of the weapon employed. Rapid identification of an unusual suicide, as opposed to a case erroneously recorded as homicide, would ensure the correct completion of related judicial procedures.^[2]^ Only scrupulous evaluation of all the elements in the investigation can reconstruct the lethal chain and solve the question about the mode of death.

## Author contributions

**Conceptualization:** Sara Lo Pinto, Francesco Ventura.

**Data curation:** Sara Lo Pinto, Rosario Barranco.

**Funding acquisition:** Francesco Ventura.

**Writing – original draft preparation:** Rosario Barranco, Fiorella Caputo, Martina Drommi.

**Writing – review and editing:** Francesco Ventura.
